# Primary Treatment Results of Nasopharyngeal Carcinoma (NPC) in Yogyakarta, Indonesia

**DOI:** 10.1371/journal.pone.0063706

**Published:** 2013-05-10

**Authors:** Maarten A. Wildeman, Renske Fles, Camelia Herdini, Rai S. Indrasari, Andrew D. Vincent, Maesadji Tjokronagoro, Sharon Stoker, Johan Kurnianda, Baris Karakullukcu, Kartika W. Taroeno-Hariadi, Olga Hamming-Vrieze, Jaap M. Middeldorp, Bambang Hariwiyanto, Sofia M. Haryana, I. Bing Tan

**Affiliations:** 1 Department of Head and Neck Oncology and Surgery, The Netherlands Cancer Institute-Antoni van Leeuwenhoek Hospital, Amsterdam, The Netherlands; 2 Department of Otorhinolaryngology, Dr Sardjito General Hospital/Faculty of Medicine, Universitas Gadjah Mada, Yogyakarta, Indonesia; 3 Department of Biometrics, The Netherlands Cancer Institute-Antoni van Leeuwenhoek Hospital, Amsterdam, The Netherlands; 4 Department of Radiotherapy, Dr Sardjito General Hospital/Faculty of Medicine, Universitas Gadjah Mada, Yogyakarta, Indonesia; 5 Division of Medical Oncology, Department of Internal Medicine, Dr Sardjito General Hospital/Faculty of Medicine Universitas Gadjah Mada, Yogyakarta, Indonesia; 6 Department of Radiation Oncology, The Netherlands Cancer Institute-Antoni van Leeuwenhoek Hospital, Amsterdam, The Netherlands; 7 Department of Pathology, VU University Medical Centre, Amsterdam, The Netherlands; 8 Department of Histology, Cell and Tumour Biology, Faculty of Medicine, Universitas Gadjah Mada, Yogyakarta, Indonesia; 9 Department of Otorhinolaryngology, Academic Medical Center (AMC), Amsterdam, The Netherlands; West Virginia University School of Medicine, United States of America

## Abstract

**Introduction:**

Nasopharyngeal Carcinoma (NPC) is a major health problem in southern and eastern Asia. In Indonesia NPC is the most frequent cancer in the head and neck area. NPC is very sensitive to radiotherapy resulting in 3-year disease-free and overall survival of approximately 70% and 80%, respectively. Here we present routine treatment results in a prospective study on NPC in a top referral; university hospital in Indonesia.

**Methods:**

All NPC patients presenting from September 2008 till January 2011 at the ear, nose and throat (ENT) department of the Dr. Sardjito General Hospital, Universitas Gadjah Mada, Yogyakarta, Indonesia, were possible candidates. Patients were included if the biopsy was a histological proven NPC without distant metastasis and were assessed during counselling sessions prior to treatment, as being able to complete the entire treatment.

**Results:**

In total 78 patients were included for treatment analysis. The median time between diagnosis and start of radiotherapy is 120 days. Forty-eight (62%) patients eventually finished all fractions of radiotherapy. The median duration of the radiotherapy is 62 days for 66 Gy. Median overall survival is 21 months (95% CI 18–35) from day of diagnosis.

**Conclusion:**

The results presented here reveal that currently the treatment of NPC at an Indonesian hospital is not sufficient and cannot be compared to the treatment results in literature. Main reasons for these poor treatment results are (1) a long waiting time prior to the start of radiotherapy, (2) the extended overall duration of radiotherapy and (3) the advanced stage of disease at presentation.

## Introduction

Cancer has become a leading cause of death and morbidity in low and middle-income countries. While this has been recognized for at least twenty years, healthcare systems in these countries often cannot deliver the prevention and care needed to overcome this challenge [1]–[4]. Most publications that address this issue are based on cancer incidence and barely report on treatment results and survival. The main reason for this is the lack of appropriate data collection in developing countries. There is a clear need for improved cancer control systems in developing country settings [5]. Recently we have introduced a Clinical Trial Data Management service (CTDMS) to monitor treatment results of Nasopharyngeal Carcinoma (NPC) in a university hospital setting in Yogyakarta, Indonesia [6].

In southern and eastern Asia NPC is a major health problem. In Indonesia NPC is the most frequent cancer in the head and neck area and ranks as the 4th most common tumour found in males. The undifferentiated histological subtype, NPC WHO III, is the most prevalent NPC type in South-East Asia and Indonesia. This type of cancer is causally associated with the Epstein-Barr virus (EBV) [7]. The incidence is estimated 6 per 100,000, leading to 12,000 new cases per year [8]–[10]. Due to the insufficient national cancer registration system, the actual number is most probably much higher. NPC is very sensitive to radiotherapy at early stage (stage I–II), but this type of treatment is not generally available for many patients in developing countries and unfortunately can induce complications after treatment if not properly administered. Since the location of the tumour has a close contact with the base of skull, the brain stem and spinal cord, radiation is hindered by dose limitations on these organs at risk. For advanced NPC (stage III–IV), standard-care is concurrent chemo radiation therapy with high-dose radiation combined with cisplatin-based regimens. This treatment approach may result in 3-year disease-free and overall survival of approximately 70% and 80%, respectively [11], [12]. A recent meta-analysis confirmed the clinical benefit of concurrent chemo radiation therapy (CCRT) compared with radiation alone (RT) in the treatment of stage III and IV NPC in endemic areas [13]. Nearly all treatment results on NPC presented in the literature derive from top-end hospitals and clinical trial settings. Here we present a prospective study on routine treatment results of NPC at a university hospital in Indonesia.

## Patients and Methods

### Patients Eligibility

All NPC patients presenting from September 2008 till January 2011 at the ear, nose and throat (ENT) department of the Dr Sardjito General Hospital, Universitas Gadjah Mada, Yogyakarta, Indonesia, were analysed. Patients were examined by CT-scan, chest X-ray, ultrasound of the abdomen and bone survey. Patients were included in the study if the biopsy was a histological proven NPC without distant metastasis and were assessed during counselling sessions prior to treatment, as being able to complete the entire treatment. Tissue analysis was performed at the pathology department of the Dr Sardjito hospital; WHO-histological classification was done according to the UICC 2002 criteria and EBV-encoded small RNA (EBER) staining was done with commercial reagents according to the manufacturer’s instructions (Dako, PNA-kit). Other inclusion criteria were measurable disease and a curative intent treatment plan. Patients were not eligible if they had NPC as a second malignancy.

### Treatment

Due to different health insurances of the patients, different treatment regimens have been administered. All patients received 66–70 Gray (Gy) (2 Gy per fraction for 5 days) external beam radiotherapy and in all cases treatment planning was 2 dimensional. The type of chemotherapy and the number of courses, and whether applied concurrent or as neo-adjuvant, was adjusted for the type of insurance and waiting time for radiotherapy at that time. Options of chemotherapy include 3–4 cycles cisplatinum-based induction chemotherapy and chemo-concurrent radiotherapy (CCRT). In induction setting either cisplatin and 5 fluorouracil (5 FU) (PF regimen) or combination of docetaxel, cisplatin and 5 FU (TPF regimen) are administered. Radiotherapy is carried out 1 month after induction chemotherapy. During the course of radiotherapy some patients also received a 6–7 cycles of weekly carboplatin AUC 2. In the setting of CCRT, a weekly 6–7 cycles of low-dose platinum (40 mg/m2) cooperated with radiotherapy. Duet o the poor physical condition of some patients with a advanced stage of disease, it was not possible to treat these patients with CCRT Rafter neoadjuvant chemotherapy.

### Therapy Assessment

Eight weeks after treatment the response was monitored by CT-scan, chest X-ray, ultrasound of the abdomen and bone survey. In case of histological proven local persistent or recurrent disease patients were offered to be treated with photodynamic therapy (PDT). In case of persistent neck lymph nodes patient were treated with a modified radical neck dissection.

### Follow Up

All patients had a regular follow-up schedule consisting of 3 monthly visits during the first two years after radiotherapy. In case of suspicion for tumour recurrence radiological examinations were performed. In case of a local or regional recurrence patients were treated with photodynamic therapy (PDT) with separate treatment of the neck.

### Photodynamic Therapy (PDT)

Drug administration of Foscan®, light administration and treatment procedures of the PDT are completely similar to and described previously in Nyst et al 2012 [14].

Patients received the dose level and the drug light interval recommended for the treatment of patients with squamous-cell carcinoma of the head and neck. These parameters are drug dose, 0.15 mg/kg Foscan®; drug-light interval: 48 or 96 hours; light dose: 20 J/cm^2^.

### Statistical Methods

The association between treatment type (CCRT vs other) and response (CR vs non-CR) was assuessed using the univariate Fisher exact test and multivariable logistic regression, adjusting for sex (male vs. female), type of insurance (poor vs. government vs. self finance) and AJCC stage (I–II vs III vs IV). In this analysis patients not receiving treatment were excluded, and patients for which no tumor assessment was available were assumed to be non-responders. A sensitivity analysis is performed excluding patients for which no tumor assessment was available. In these analyses the level of significance was set at 0.05. Overall (OS) and disease free survival (DFS) durations were calculated from date of diagnosis of NPC. For OS duration was until date of death from any cause or date, while for DFS duration was until development of recurrence or death from any cause. In both cases in the absence of an event patients were censored at the date of last contact. The Kaplan-Meier technique was used to estimate survival. All analyses were performed using the R-software version 2.15.2.

## Results

In total 188 patients presented with NPC during September 2008 till January 2011. Seventy-eight patients were included for treatment analysis, as they were considered able to complete treatment protocol based on intake counselling. The median age of the patients was 49 years (range 17–78), 50 patients were male and 28 were female patients. Only 2 patients had early stage disease at entry being stage I or IIA, and 76 patients presented with loco-regional advanced stage IIB and higher. All 78 patients had an EBER-positive, histological proven NPC and were WHO type III. Reasons for exclusion were insufficient funding to afford a curative treatment (n = 27; 25%), no sufficient funding to afford staging procedures (n = 24, 22%), no pathology available (n = 24, 22%), decided to go for traditional treatments (n = 16, 15%), eleven patients presented with distant metastasis at diagnosis (n = 11, 10%), six patients due to logistic reasons (7%) and two refused the protocol (2%). Patient characteristics are listed in table 1. Types of chemotherapy regimen administered are listed in table 2.

**Table 1:** Patient characteristics and type of treatment.

	None*	Concurrent+RT	Neo Adjuvant+Concurrent+RT	Neo Adjuvant+RT	RT	All
	N = 8	N = 18	N = 9	N = 41	N = 2	N = 78
**Age**						
Median	48	49	45	51	38	49
(Range)	(41–72)	(17–73)	(27–54)	(23–78)	(29–47)	(17–78)
**Sex**						
F	1 (12%)	8 (44%)	2 (22%)	16 (39%)	1 (50%)	28 (36%)
M	7 (88%)	10 (56%)	7 (78%)	25 (61%)	1 (50%)	50 (64%)
**T stage**						
T1	2 (25%)	2 (11%)	1 (11%)	10 (24%)	1 (50%)	16 (21%)
T2A	1 (12%)	2 (11%)	0 (0%)	5 (12%)	0 (0%)	8 (10%)
T2B	0 (0%)	4 (22%)	1 (11%)	5 (12%)	1 (50%)	11 (14%)
T3	1 (12%)	7 (39%)	7 (78%)	17 (41%)	0 (0%)	32 (41%)
T4	4 (50%)	3 (17%)	0 (0%)	4 (10%)	0 (0%)	11 (14%)
**N stage**						
N0	0 (0%)	1 (6%)	2 (22%)	5 (12%)	1 (50%)	9 (12%)
N1	3 (38%)	6 (33%)	1 (11%)	4 (10%)	1 (50%)	15 (19%)
N2	0 (0%)	8 (44%)	2 (22%)	12 (29%)	0 (0%)	22 (28%)
N3A	2 (25%)	3 (17%)	3 (33%)	15 (37%)	0 (0%)	23 (29%)
N3B	3 (38%)	0 (0%)	1 (11%)	5 (12%)	0 (0%)	9 (12%)
**AJCC stage**						
	0 (0%)	0 (0%)	0 (0%)	0 (0%)	1 (50%)	1 (1%)
IIA	0 (0%)	0 (0%)	0 (0%)	1 (2%)	0 (0%)	1 (1%)
IIB	0 (0%)	3 (17%)	1 (11%)	1 (2%)	1 (50%)	6 (8%)
III	0 (0%)	9 (50%)	4 (44%)	15 (37%)	0 (0%)	28 (36%)
IVA	3 (38%)	3 (17%)	0 (0%)	3 (7%)	0 (0%)	9 (12%)
IVB	5 (62%)	3 (17%)	4 (44%)	21 (51%)	0 (0%)	33 (42%)
**Type of insurance**						
Government Insurance	0 (0%)	1 (6%)	3 (33%)	1 (2%)	1 (50%)	6 (8%)
Poor Insurance	7 (88%)	17 (94%)	3 (33%)	39 (95%)	0 (0%)	66 (85%)
Self Finance	1 (12%)	0 (0%)	3 (33%)	1 (2%)	1 (50%)	6 (8%)

* The 8 patients who haven’t received any treatment died before the start of treatment.

**Table 2:** Type of chemotherapy administered.

	Concurrent+RT	Neo Adjuvant+Concurrent+RT	Neo Adjuvant+RT	All
	N = 18	N = 9	N = 40	N = 78
**Neoadjuvant chemotherpy**				
Carboplatin, 5 FU			2 (5%)	2 (3%)
Cisplatin, 5 FU			36 (88%)	36 (46%)
Docetaxel, Cisplatin, 5 FU		9 (100%)	1 (2%)	10 (13%)
Paclitaxel, Cisplatin			1 (2%)	1 (1%)
**Concurrent chemotherapy**				
Carboplatin	1 (6%)	7 (78%)		8 (10%)
Cisplatin	17 (94%)	1 (11%)		18 (23%)

### Radiotherapy

The median time between diagnosis and start of radiotherapy is 120 days (range 13–500). Twelve (15%) patients died before the start of radiotherapy due to disease progression or in two cases due to side effects of the neo-adjuvant chemotherapy treatment. Four patients did not return for radiotherapy due to concern about the side effects after the neo-adjuvant chemotherapy and two patients had, despite counselling, insufficient funding to be treated with radiotherapy.

Forty-eight (62%) patients eventually finished all fractions of radiotherapy. The median duration of the radiotherapy is 62 days (range 46–140 days) for 66 Gy.

### Response to Treatment

Twenty three (29%) patients died of disease progression before treatment response could be assessed. Five patients dropped out due to insufficient funding, 5 stopped treatment early due to side effects and 6 stopped treatment due to concern for side effects. Eventually 39 (50%) patients had a treatment response measurement 8 weeks after treatment. Twenty-three (29%) achieved a complete response. Ten patients experienced local persistent disease, in two patients regional persistent disease was found and two patients developed distant metastasis. Two patients had local and regional persistent disease, one of them also developed distant metastasis. The improvement in response rate after receiving concurrent chemo-radiotherapy was significant (p = 0.002). Adjusting for sex, insurance type and AJCC stage did not change this result (p = 0.001), nor did the exclusion of patients non-evaluable for response (univariate p = 0.02; multivariable p = 0.02).

### Survival

**Figure 1:**
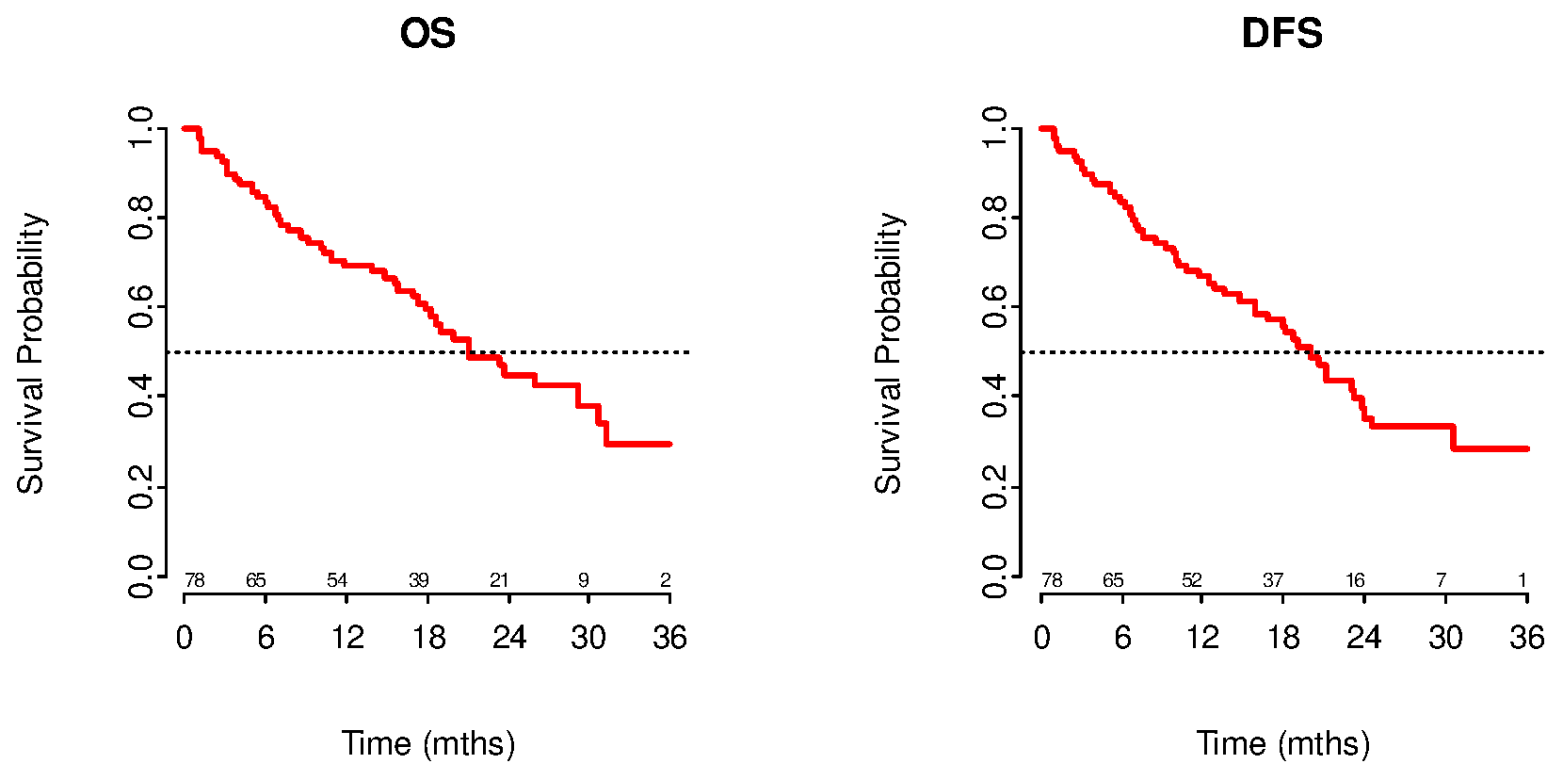
Kaplan Meijer curves present the survival probability for overall survival and disease free survival respectively.

Median overall survival is 21 months (95% CI: 18–35) from day of diagnosis. The median disease free survival from day of diagnosis is 20 months (95% CI: 18–24). In figure 1A and figure 1B Kaplan Meijer curves present the survival probability for overall survival and disease free survival respectively.

### Photodynamic Therapy

Six patients with local persistent disease, which was discovered during therapy assessment after fulfilment of the regular treatment, and one patient with a local recurrence, discovered during follow up, has been treated with PDT. All patients had a complete response 12 weeks after illumination, three patients developed regional recurrences and one patient died due to the regional recurrence.

## Discussion

The results presented here reveal that currently the treatment of NPC at an Indonesian academic hospital is not sufficient and cannot be compared to the treatment results in literature [11], [12]. Of the patients evaluable for response only 29% had a complete response directly after treatment, with the median overall survival being 21 months after diagnosis. Main reasons for these poor treatment results are (1) a long waiting time prior to the start of radiotherapy, (2) the extended overall duration of radiotherapy and (3) the advanced stage of disease at presentation.

Cancer is becoming a growing problem in low and middle-income countries. Since it is ill-defined how many of the patients actually make it to the hospital and receive a biopsy that is registered, the actual NPC incidence remains unclear. NPC is the commonest head and neck cancer in Indonesia with most patients presenting with an advanced stage of disease which poses a heavy burden on the population. A major problem is that patients cannot afford treatment or are afraid of treatment toxicity, causing them to refrain from treatment or seek alternative options. This resulted in nearly 61% of the excluded NPC patients being non-compliant with the inclusion criteria for this study. However, the NPC treatment results presented here reveal an additional problem, this being the limited capacity for proper treatment. In 2008 there were 18 linear accelerators and 17 Cobalt-60 teletherapy machines available in Indonesia with a population of 229 million, nevertheless 6 of them were under commission. Resulting in 0.13 accelerator per million inhabitants [15]. This in comparison to Europe were 5.5 accelerator is available per million inhabitants in the high, 3.5 per million in the medium and 2 per million in the low resource countries [16]. The recommended number of treatment units per population differs widely, in Europe guidelines of 25 low to high income countries recommended on average 5.9 per million [17].

Although Indonesia is a rising economy, meeting these criteria will not be feasible on short term. Expanding radiotherapy facilities is a time and money-consuming project. For building a new radiotherapy facility and have it installed for treatment the average time is approximately 5 years. Expansion of staffing (radiation oncologists, physicists, technologists) takes even longer, since proper education and training is necessary [16].

When patients with poor insurance in Yogyakarta finally complete diagnosis and imaging to stage the disease, they have to wait on average 4 months to start radiotherapy treatment. At this time 13% have already succumbed to disease progression. Despite optimal counselling in this study cohort, five patients who started treatment did not finish the treatment properly because they could not afford the costs of the entire treatment. Importantly, we noted that most patients who eventually started treatment received insufficient radiotherapy treatment. Optimally, a total dose of 66 to 70 Grey should be given in 33 to 35 fractions and the best therapy response is achieved when the total dose is administered in 45 to 47 days. Every day the radiotherapy is postponed a loss of the effective dose occurs. This will influence the treatment success to a great extent [18], [19].

The median duration of radiotherapy treatment in this study was 62 days. This prolonged treatment time together with the 2-dimensional radiotherapy technique based on CT imaging only (i.e. not using MRI to assess disease extent) are the main causes of the poor response rate [20], [21]. In part this prolonged treatment duration is often caused by the radiotherapy facility being intermittently operational, due to poor maintenance of the equipment. Other causes for this delay may be due to poor efficiency in administration and communication at and between hospital departments and poor patient and doctor compliance to protocols and timelines. Further research is required to reveal these causes and can eventually contribute to improve overall treatment time.

The late stage of NPC at presentation in the hospital is another unfavourable prognostic factor. One possible reason for the high percentage of patients with advanced NPC could be due to poor diagnosis by general practitioners (GP) and thereby a delay in referral. In our previous study we assessed the knowledge on NPC of the GPs working in the Primary Health Care Centres in the Yogyakarta region [22]. Our results indicate that the knowledge of GPs is insufficient, with many of them not being aware of the high incidence of NPC in their region.

In Malaysia prior studies have proved that the lack of awareness and knowledge of primary health care workers is one of the main reasons for delayed diagnosis [23]. Given that presenting stage is the most important prognostic factor, appropriate training of GPs is critical. The relevance of adequate referral by GPs for head and neck carcinomas has been shown by Alho et al. [24], who found that 20% of 221 patients, subsequently to being diagnosed with head and neck carcinoma, were initially sent home without referral. The risk of death in this group was significantly higher when compared with the patients who were immediately referred or received a follow up appointment.

The same group from Finland, has also shown that the time between GP referral and final diagnosis is a significant factor in patient outcome in other head and neck cancer [25]. Long delay in primary care resulted in a significantly worse prognosis in patients with laryngeal carcinoma [26].

All patients analysed in the presented study had WHO type III positive NPC. Prior studies have shown that EBV-related markers can be used for screening and prognostic monitoring. These markers include EBV (IgA) serology and EBV-DNA load in nasopharyngeal brushings or blood. NPC patients have characteristic aberrant IgG and IgA antibody reactivity to several EBV encoded antigens as well as increased EBV-DNA in blood plasma, derived from shed (apoptotic) tumour fragments into the circulation. Increased IgA antibody levels are found against early antigen (EA), viral capsid antigen (VCA) and the latent Epstein-Barr nuclear antigen 1 (EBNA1) as well as inhibitory antibodies to the EBV specific DNAse [27], [28]. These antibody responses against defined viral antigens are the basis of a proposed screening test for NPC in high-risk populations [29]–[31]. Recent insight in the molecular basis and diversity of anti EBV IgA and IgG responses allowed the development of more defined serological tools [32]–[36]. Importantly, such initial NPC-risk analysis can be done in the regional hospital setting with small volumes of blood, collected by finger prick sampling on filter paper, thus providing a cheap approach [35], [37]. The fingerpick sample might also allow EBV-DNA load measurement, since DNA is a rather stable molecule in dried blood. Furthermore, the nasopharyngeal brushing with EBV marker assessment, may provide a promising method for detecting tumour presence in situ, by measuring EBV-DNA and RNA in parallel. Such brushings can be taken with simple tools under nasal-endoscope guidance [38]–[42].

Future education programs should include referencing to the available improved EBV-based diagnostic procedures for NPC-risk screening and early detection. Improved education combined with a screening method could be a cheap and sensitive screening method for NPC in Indonesia and other high incidence countries. Application of these methods in patients with chronic complaints in the head and neck, not responding to traditional antibiotic and anti-allergic medication has already yielded successful detection of early stage NPC cases in the Sardjito hospital patient population [43].

Currently we enrolled NPC awareness programme in Jakarta, Yogyakarta and Surabaya. Short time efficacy of the training programmes have been published recently [44]. Hopefully in the future this programme can be expanded to include more regions of Indonesia.

The patients treated with PDT participate in an ongoing phase II trial, the results on PDT treatment for persistent and recurrent disease will be presented in more detail when the study has been completed. Still worth mentioning is that out of the seven patients who were treated with PDT, six of them are still alive. This is 17 per cent of all included NPC patients who are still alive. Based on our experiences and taking into account the poor treatment results currently for primary NPC in Indonesia, we assume that PDT can successfully be used in the future as part of the primary treatment. We hypothesize that PDT can be applied for NPC during the waiting time for radiotherapy, without compromising the options for all other possible treatment modalities. Perhaps the physical condition of the patients remains in such a good condition that at time the treatment can start patient can receive CCRT, which showed to have the best improved response rate.

The easiest ways of addressing current problems would be establishing sufficient radiotherapy facilities in Indonesia, however this will take decades. In the mean while our goal has to be to assure that patients who will be treated have an effective treatment and the right candidates are selected. Early detection of NPC, new treatment regimens to overcome the waiting time, a counselling system that supports only those patients who have a real opportunity to complete the treatment and improved maintenance for the radiotherapy facility are feasible short-term solutions to improve treatment outcome in the near future.
